# Uptake of Tyrosine Amino Acid on Nano-Graphene Oxide

**DOI:** 10.3390/ma11010068

**Published:** 2018-01-04

**Authors:** Hossam M. Nassef, Mohamed Hagar, Zeiad Malek, Abdelhameed M. Othman

**Affiliations:** 1 Chemistry Department, Faculty of Science at Yanbu, Taibah University, Yanbu 46423, Saudi Arabia; hossamnassef2002@gmail.com (H.M.N.); mohamedhaggar@gmail.com (M.H.); waleed2993@gmail.com (Z.M.); 2 Chemistry Department, Faculty of Science, Damietta University, Damietta 34517, Egypt; 3 Chemistry Department, Faculty of Science, Alexandria University, Alexandria 23132, Egypt; 4 Environmental Biotechnology Department, Genetic Engineering and Biotechnology Research Institute, University of Sadat City, Sadat City 32897, Egypt

**Keywords:** graphene oxide, amino acid, tyrosine, uptaking, SEM

## Abstract

Graphene oxide (GO) is emerging as a promising nanomaterial with potential application in the detection and analysis of amino acids, DNA, enzymes, and proteins in biological fluid samples. So, the reaction of GO with amino acids should be characterized and determined before using it in biosensing methods and devices. In this study, the reaction of tyrosine amino acid (Tyr) with GO was characterized using FT-IR, UV-vis spectrophotometry, and scanning electron microscopy (SEM) before its use. The optimum conditions for GO’s interaction with Tyr amino acid have been studied under variable conditions. The optimum conditions of pH, temperature, shaking time, and GO and tyrosine concentrations for the uptaking of tyrosine amino acid onto the GO’s surface from aqueous solution were determined. The SEM analysis showed that the GO supplied was in a particle size range between 5.4 and 8.1 nm. A pH of 8.4–9.4 at 25 °C and 5 min of shaking time were the optimum conditions for a maximum uptake of 1.4 μg/mL of tyrosine amino acid onto 0.2 mg/mL of GO.

## 1. Introduction

Graphene oxide (GO) is a novel single-atom-thick and two-dimensional carbon material produced by the oxidation of graphite that contains oxygen functional groups, such as epoxides, phenol hydroxyls, and carboxylic groups [1,2,3]. The oxygenated lattice of GO not only facilitates better water solubility and stability, but allows for noncovalent interaction with diols, amine functional groups, and phenyls in biomolecules through electrostatic interaction, π–π stacking, and hydrogen bonding to enable the recognition of biomolecules with detectable specificity [4]. Recently, GO has attracted considerable attention due to its extraordinary electronic, optical, and thermal properties in comparison to other nanomaterials. The superior characteristics of GO, such as its large surface area, good water dispersibility and biocompatibility, facile surface modification, and low manufacturing cost, make it a promising material for biotechnology and biosensing applications [5,6,7,8]. GO has been shown to quench organic fluorescence molecules due to long-range nano-scale energy transfer [9].

Recent experimental results indicate that some amino acids, peptides, and proteins can be quickly adsorbed onto the surface of GO because of electrostatic interaction, hydrophobic interaction, and hydrogen bonding [10]. Amino acids are molecules containing at least one amino group, a carboxylic group, and a side chain that varies between different amino acids. Amino acids are important to life and have many functions in metabolism; one particularly important function is to serve as the building blocks of proteins. Many proteins have residual amino acids in their structure, such as tryptophan and tyrosine (Tyr), and due to their central role in biochemistry, amino acids are important in nutrition and are commonly used in food technology and industry. Tyrosine and Tryptophan are two amino acids that contribute to our emotional well-being and mental alertness as well as participate in a wide variety of other healthful benefits [11]. Amino acids appear as promising molecules to be immobilized on nanomaterials [12,13,14]. One of the recent studies in this area used GO as an optical sensor for the determination of the L-tryptophan amino acid (Trp) in the presence of other amino acids, such as L-asparagine, L-histidine, L-phenylalanine, L-arginine, L-tyrosine, L-lysine, L-ascorbic acid, tartaric acid and uric acid, by a spectrofluorometric method [15]. The chemical composition of GO is not yet fully known, and due to the high reactivity of the oxygenated groups, mainly the epoxy, hydroxyl, and carboxyl groups, several derivatization reactions may occur at the same time [16]. In this study, graphene oxide was used as an absorbing material for tyrosine amino acids in aqueous solution. Different parameters were tested to determine the optimum conditions for the uptaking of tyrosine amino acid, such as pH, temperature, GO and tyrosine concentration, and shaking time. These data showed that the uptake of tyrosine amino acid on the surface of GO can serve as a technique for the separation and determination of amino acids or protein with residual tyrosine amino acid from aqueous and biological fluids.

## 2. Experimental

### 2.1. Material and Instruments

Graphene oxide was obtained from Advanced Chemical Supplier ACS Material LLC., Pasadena, CA, USA. The particle size of the GO was determined by Scanning Electron Microscopy (SEM-FEI, FEG Quants 250, Hillsboro, OR, USA, magnification 20–1,000,000× high resolution). All spectrophotometric measurements were carried out at 25 ± 1 °C using a UV-vis spectrophotometer model (UV-1800 SHIMADZU, Nakagyo-KU, Kyoto, Japan). Fourier Transform Infrared Spectroscopy (FTIR) was carried out using a Nicolet iS 10 Thermo scientific (Waltham, MA, USA). A bench-top M545 meter was applied to adjust the pH (Pinnacle, Corning, NY, USA). A bench-top sonicator of Model 1510 was used to disperse graphene oxide in water and for the shaking time experiments (Branson, Danbury, CT, USA). The tyrosine amino acids were delivered from Sigma Co. (Munich, Germany).

### 2.2. Methods

#### 2.2.1. Particle Size Determination of GO

The shape and size of the GO were studied by scanning electron microscopy (SEM-FEI, FEG Quants 250, Hillsboro, OR, USA, magnification 20–1,000,000× high resolution).

#### 2.2.2. FT-IR of GO

Fourier Transform Infrared Spectroscopy (FTIR) with a Nicolet iS 10 Thermo scientific (Waltham, MA, USA) was used to record and characterize the spectra of the GO before uptake within a scanning range of 400–4000 cm^−1^.

#### 2.2.3. UV-vis of GO

UV-vis spectral data were measured on a spectrophotometer (UV-1800 SHIMADZU, Nakagyo-KU, Kyoto, Japan) in an aqueous solution of GO, Tyr, and GO–Tyr. The maximum absorption of wavelength was 279 nm for Tyr and 223 nm for GO, and the absorption spectra and area under peak were measured and calculated for different variables at 274 nm.

#### 2.2.4. Effect of GO Concentration

A stock solution of 1 mg/mL of GO aqueous dispersion was obtained by adding 50 mL of pure water to 50 mg of GO, followed by sonication for 1 h in a cold water bath and dilution with pure water according to the needs of the experiment. Different concentrations (0.1, 0.2, 0.3, and 0.4 mg/mL) of GO were prepared. One milliliter of each GO concentration was added to 4.0 mL of 1 × 10^−4^ M of Tyr, then the absorption spectra of Tyr–GO were recorded and the area under peak was calculated.

#### 2.2.5. Effect of pH

Measurements of the pH dependence of 4.0 mL of 1 × 10^−4^ M Tyr amino acid over 1 mL of GO (1 mg/mL) solution were performed over a wide range of pH (3–11). The pH was adjusted using dilute sodium hydroxide and/or hydrochloric acid, then the tested solution was completed to 5 mL with pure water. The absorption spectra and the area under peak were measured and calculated with different pHs at 274 nm.

#### 2.2.6. Effect of Temperature

The effect of temperature on the reaction of Tyr amino acid with GO was done by adding 4.0 mL of 1 × 10^−4^ M of Tyr amino acid solution to 1 mL of GO (1 mg/mL) solution and completing the solution to 5 mL by pure water. The absorption spectra and the area under peak were measured and calculated at different temperatures: 20, 25, 30, 35, and 40 °C at 274 nm.

#### 2.2.7. Effect of Shaking Time

The effect of time, with and without shaking, on the uptake of Tyr amino acid on the GO’s surface has been investigated. Four milliliters of 1 × 10^−4^ M Tyr solution was added to 1 mL of GO solution (1 mg/mL). The absorption spectra were measured at 0.0, 5, 10, 15, 20, and 25 min at 25 ± 1 °C and the area under peak at 274 nm was calculated.

#### 2.2.8. Uptake of Tyr Amino acid on GO

To study the uptake of Tyr amino acid on the surface of GO, a solution of 4.0 mL of 1 × 10^−4^ M Tyr and 1 mL of GO (1 mg/mL) was prepared. The absorbance of the Tyr, GO, and Tyr/GO solutions was measured, and the areas under peak were calculated.

## 3. Results and Discussion

Using graphene oxide (GO) in biosensing and devices has recently been of significant interest in the biomedical field for the detection of specific biomolecules from body fluid samples [17,18,19,20]. GO has attracted considerable attention due to its extraordinary electronic, optical, and thermal properties in comparison to other nanomaterials. The superior characteristics of GO, such as its large surface area, good water dispersibility and biocompatibility, facile surface modification, and low manufacturing cost, make it a promising material for biotechnology and biosensing applications [21,22].

The reaction of GO with amino acids is interesting and has drawn attention in the last few years due to the high reactivity of the oxygenated moieties, mainly the epoxy, hydroxyl, and carboxyl groups [14].

The aim of our study is to characterize and determine the optimum conditions for the uptake of Tyr amino acid onto the surface of GO.

### 3.1. Characterization of GO 

A commercially available GO, used in this study, was not synthesized in our lab. It was purchased from Advanced Chemical Supplier (ACS) Material LLC (Medford, MA, USA), and has been fully characterized by the company [23] (supplementary data, Figures S1–S6).

#### 3.1.1. FT-IR of GO

**Figure 1:**
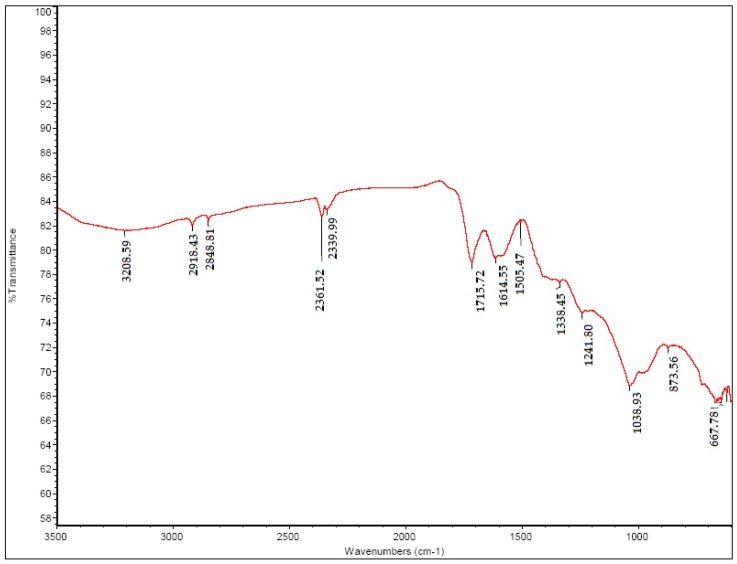
FT-IR of graphene oxide (GO).

The FT-IR of GO (Figure 1) showed a strong peak at 1715 cm^−1^ assigned to C=O (carbonyl/carboxy), while the peak at 1614 cm^−1^ was attributed to C=C (aromatics), the peak at 1437 cm^−1^ C–O (carboxy), the peak at 1241 cm^−1^ C–O (epoxy), and the peak at 1038 cm^−1^ C–O (alkoxy). These data are consistent with previously reported studies [17,18,19,20,22].

#### 3.1.2. UV-vis of GO

**Figure 2:**
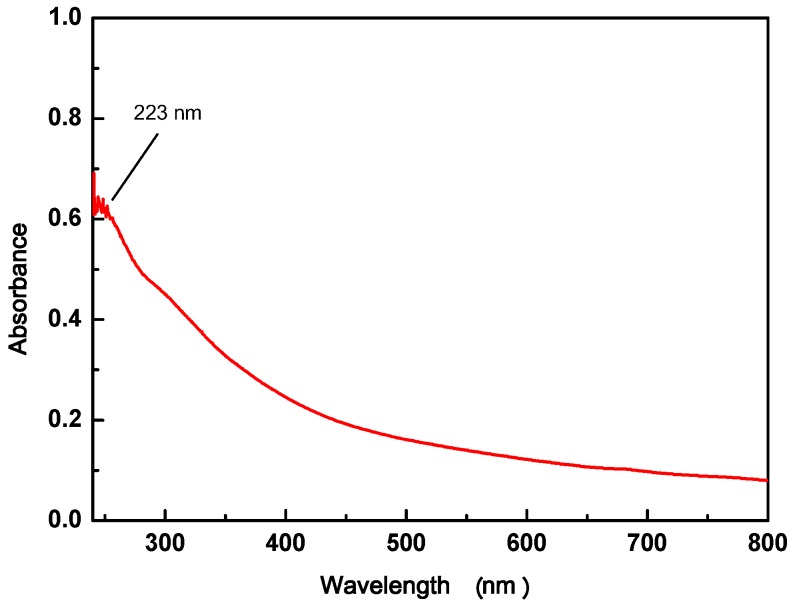
UV-vis of GO.

The UV-vis spectra of GO are shown in Figure 2. They exhibit a maximum absorption peak at about 223 nm, corresponding to the π–π* transition of aromatic C–C bonds.

#### 3.1.3. SEM of GO

**Figure 3:**
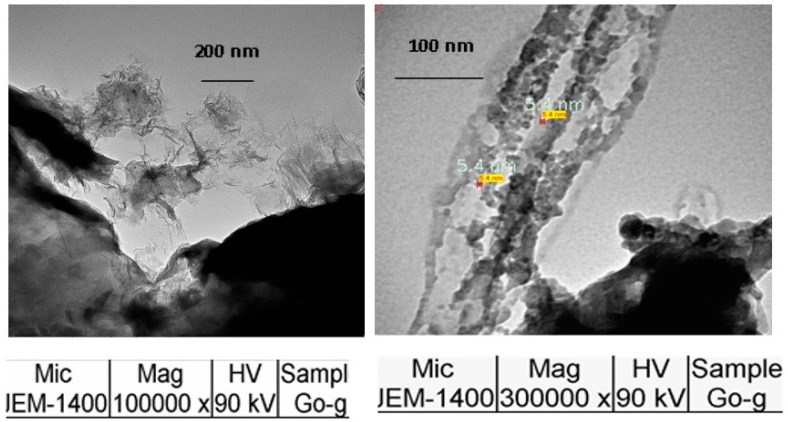
SEM analysis of GO.

Morphological studies of the GO were performed by SEM. The SEM micrographs are shown in Figure 3. It can be observed that the matrix of GO is clear and that the particle size is in the range of 5.4 to 8.1 nm. The particles are regular in shape and not variable in size.

### 3.2. Effect of GO Concentration

**Figure 4:**
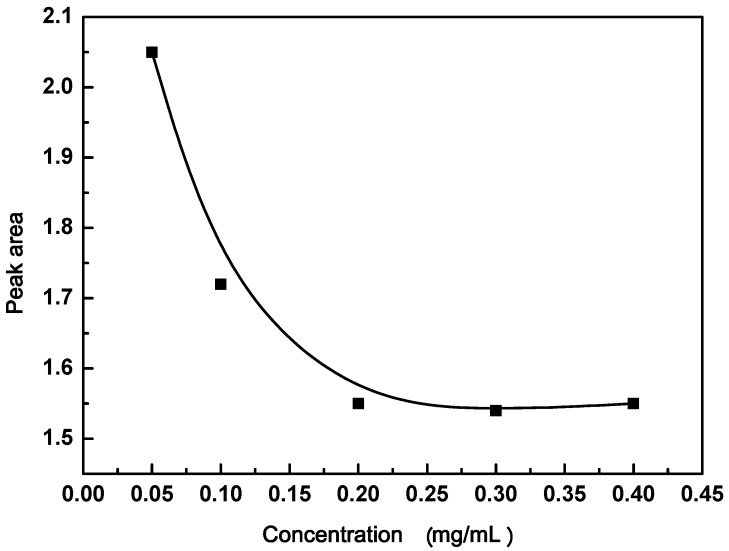
The influence of GO concentration on the adsorption of tyrosine (Tyr) amino acid.

The uptake of Tyr in the presence of different concentrations (0.05, 0.1, 0.2, 0.3, and 0.4 mg/mL) of GO is shown in Figure 4. The calculated data of the corresponding peak area showed that the minimum concentration of GO used for the maximum uptake of Tyr amino acid was 0.2 mg/mL. So, this concentration was used in the subsequent experiments.

### 3.3. Effect of pH

**Figure 5:**
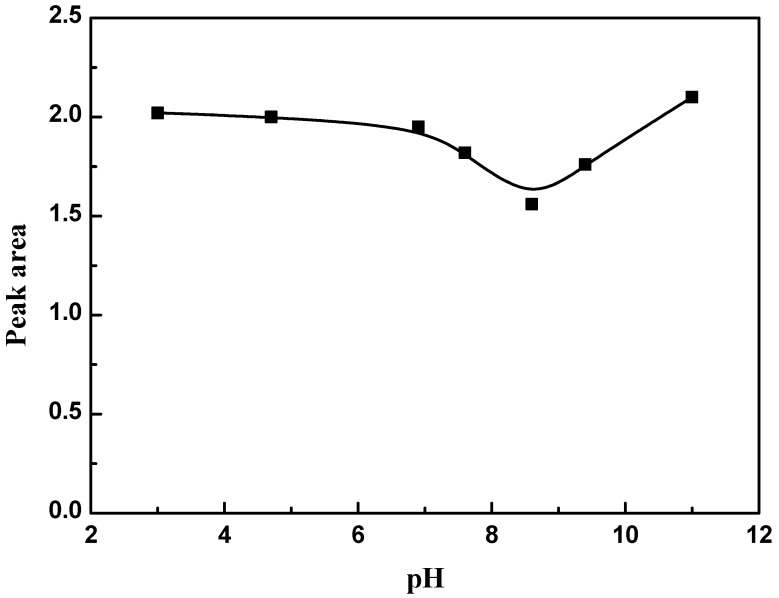
Effect of pH on the adsorption of Tyr amino acid on the GO’s surface.

Different pHs of Tyr–GO solutions (3.0, 4.7, 6.9, 7.6, 8.6, 9.4, and 11.0) were prepared, and the absorption spectra and the area under peak were calculated to determine the optimum pH for the adsorption of Tyr onto GO. The obtained data (Figure 5) showed that the smallest area under peak was found at a pH between 8.4 and 9.4. These data indicate that the interaction between Tyr and GO was increased when increasing the pH up to pH 8.6, then the peak area returned to an increase. This adsorption behavior of Tyr is mainly due to an electrostatic attraction to GO, so as the pH increases, the negative charge on Tyr increases and consequently the uptake of Tyr on the GO’s surface decreases.

### 3.4. Effect of Temperature

The absorption spectra were recorded at different temperatures (20, 25, 30, 35, and 40 °C), and each corresponding peak area was calculated. The data presented in Table 1 show that changing the temperature has no significant impact on the adsorption of Tyr. Therefore, the preferred temperature for the adsorption of Tyr onto the GO’s surface is 25 °C.

### 3.5. Effect of Shaking Time

**Figure 6:**
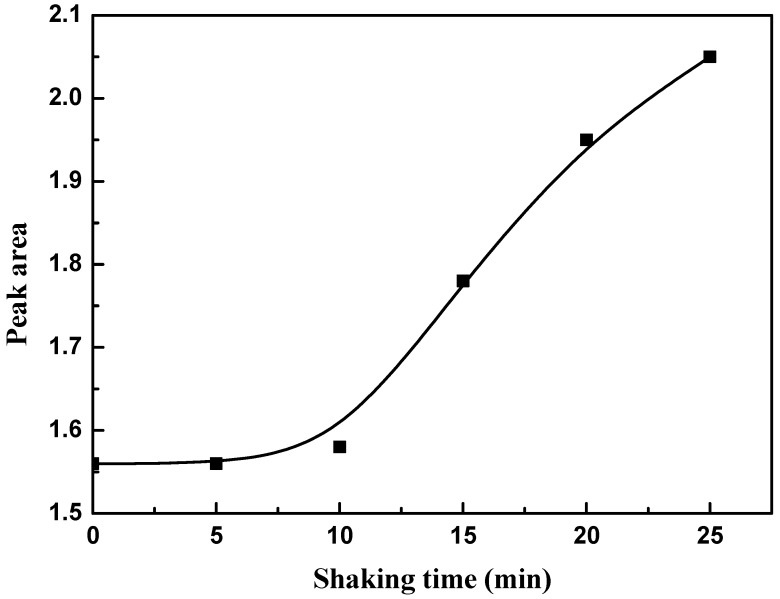
Effect of shaking time on the adsorption of Tyr amino acid on GO.

The effect of shaking time at 25 °C was investigated (Figure 6), and the data obtained showed that the adsorption of Tyr on the GO’s surface occurred immediately and stably up to 10 min without the necessity of shaking. For a shaking time greater than 10 min, the area under peak was increased and this could be explained in terms of releasing Tyr from the GO’s surface.

### 3.6. Adsorption of Tyr Amino Acid on GO Surface

**Figure 7:**
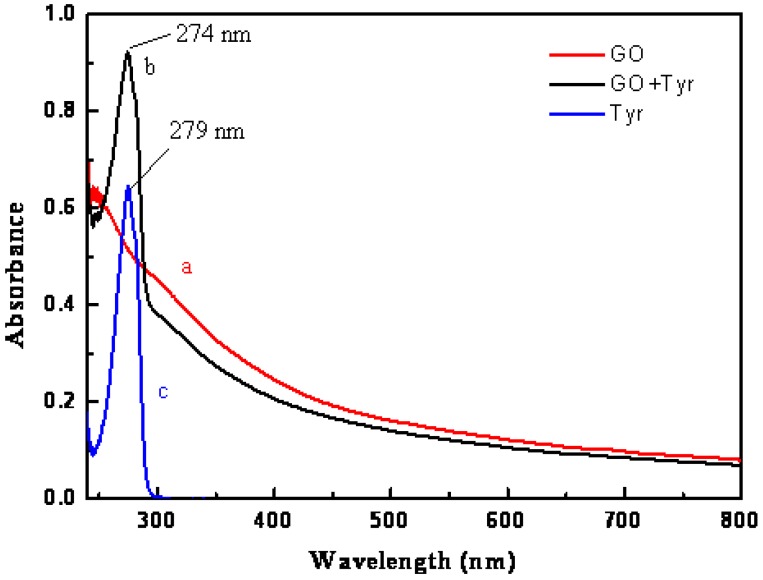
UV-visible absorption spectra for (**a**) GO; (**b**) GO in the presence of Tyr amino acid; and (**c**) Tyr amino acid.

Under the optimum condition (1 mL of GO stock solution was added to 4 × 10^−4^ M of Tyr at 25 °C, with the pH adjusted to 8.4) of the variables previously discussed, comparative UV-vis spectra were recorded. The area under peak was calculated after 5 min. As shown in Figure 7, the maximum absorbance of tyrosine appeared at 279 nm and after interaction of Tyr with the GO’s surface, the peak was shifted to 274 nm (by a 5 nm decrease) and the peak of Tyr was carried on the GO peak. Also, the area under peak was decreased from 2.18 to 1.56. These data indicated that Tyr was well-adsorbed on the surface of the GO.

## 4. Conclusions

The adsorption of Tyr on the surface of GO was characterized and determined. The SEM analysis showed that the particle size of the GO used in this study ranged between 5.4 and 8.1 nm. The UV-vis data and FT-IR indicated that the GO was on the nano-scale. The experimental data showed that Tyr was adsorbed rapidly at 25 °C and that the suitable concentration of GO is 0.2 mg/mL for 8 × 10^−5^ M of Tyr amino acid at pH 8.4. The mechanism of adsorption of Tyr is mainly electrostatic attraction as it is clear from the study of the pH’s effect. The maximum absorbance of tyrosine was obtained after its interaction with the GO’s surface. The peak was shifted to 274 nm (by a 5 nm decrease), and the peak of Tyr overlapped the GO’s peak. Also, the area under peak was decreased from 2.18 to 1.56. These data indicate that Tyr is well-adsorbed on the surface of GO.

## Figures and Tables

**Table 1:** Effect of temperature on the adsorption of Tyr amino acid on GO.

Temperature (°C)	Peak Area (A)
20.0	1.57
25.0	1.56
30.0	1.56
35.0	1.58
40.0	1.56

## Supplementary Materials

The following are available online at www.mdpi.com/1996-1944/11/1/68/s1, Figure S1: TEM image of single layer graphene (ACS Material-Graphene Factory), Figure S2: SEM image of single layer graphene (ACS Material-Graphene Factory), Figure S3: HRTEM image of single layer graphene (ACS Material-Graphene Factory), Figure S4: XRD patterns of single layer graphene (ACS Material-Graphene Factory), Figure S5: XPS patterns of single layer graphene (ACS Material-Graphene Factory), Figure S6: Raman spectrum of single layer graphene (ACS Material-Graphene Factory).
