# Supplementary material for: Uptake of Tyrosine Amino Acid on Nano-Graphene Oxide
Source: Materials (Basel). 2018 Jan 4;11(1):68. doi: 10.3390/ma11010068 (PMC5793566; doi:10.3390/ma11010068)
Supplement: Supplementary file 1 [file materials-11-00068-s001.pdf]

Supplementary data

Uptake of Tyrosine Amino Acid on Nano-Graphene Oxide

Hossam M. Nassef [1,2], Mohamed Hagar [1,3], Zeiad Malek [1], and Abdelhameed M. Othman [1,4,*]

[1] Chemistry Department, Faculty of Science at Yanbu, Taibah University, Yanbu 46423, Saudi Arabia; hossamnassef2002@gmail.com (H.M.N.); mohamedhaggar@gmail.com (M.H.); waleed2993@gmail.com (Z.M)
[2] Chemistry Department, Faculty of Science, Damietta University, Damietta 34517, Egypt
[3] Chemistry Department, Faculty of Science, Alexandria University, Alexandria 23132, Egypt
[4] Environmental Biotechnology Department, Genetic Engineering and Biotechnology Research Institute, University of Sadat City, Sadat City 32897, Egypt
* Correspondence: ayosefaly@taibahu.edu.sa; Tel.:+966-540-673-967

GO used in this study was purchased from Advanced Chemical Supplier (ACS) Material LLC. It has been fully characterized by the company [23] as mentioned below:

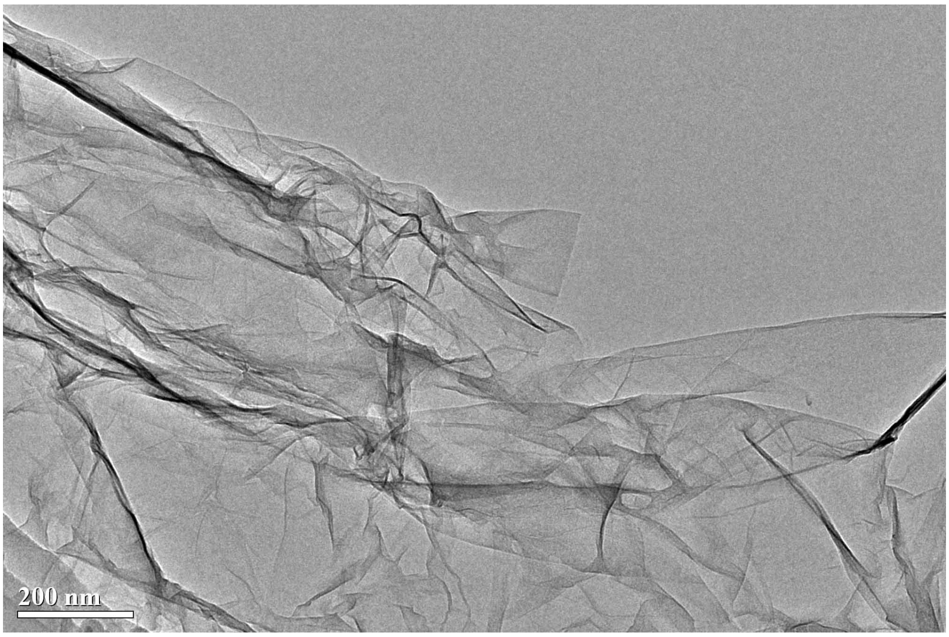

Figure S1. TEM Image of Single Layer Graphene (ACS Material-Graphene Factory)

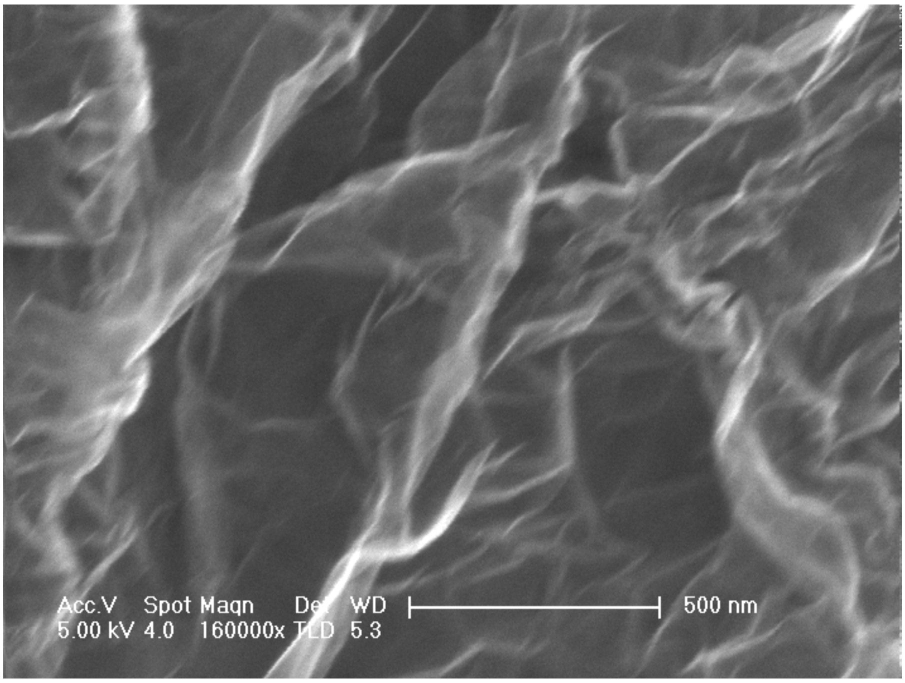

Figure S2. SEM Image of Single Layer Graphene (ACS Material-Graphene Factory)

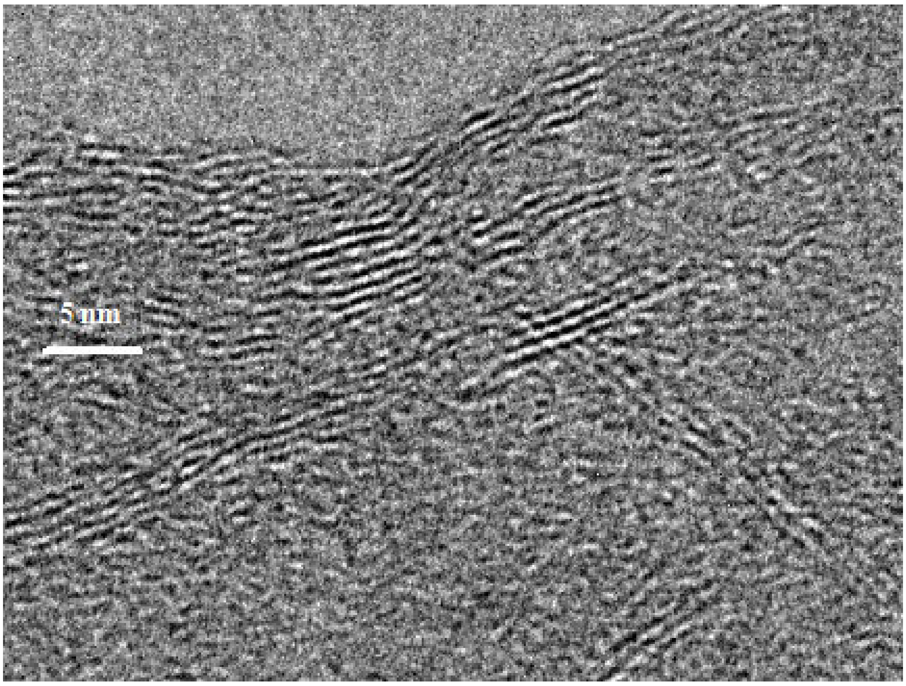

Figure S3. HRTEM Image of Single Layer Graphene (ACS Material-Graphene Factory)

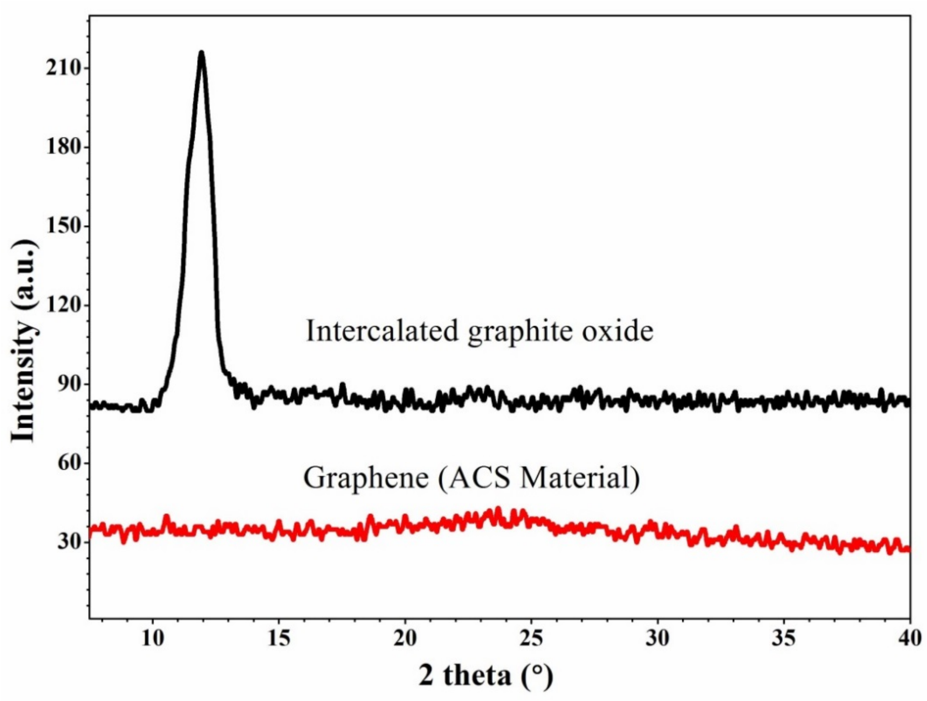

Figure S4. XRD Patterns of Single Layer Graphene (ACS Material-Graphene Factory)

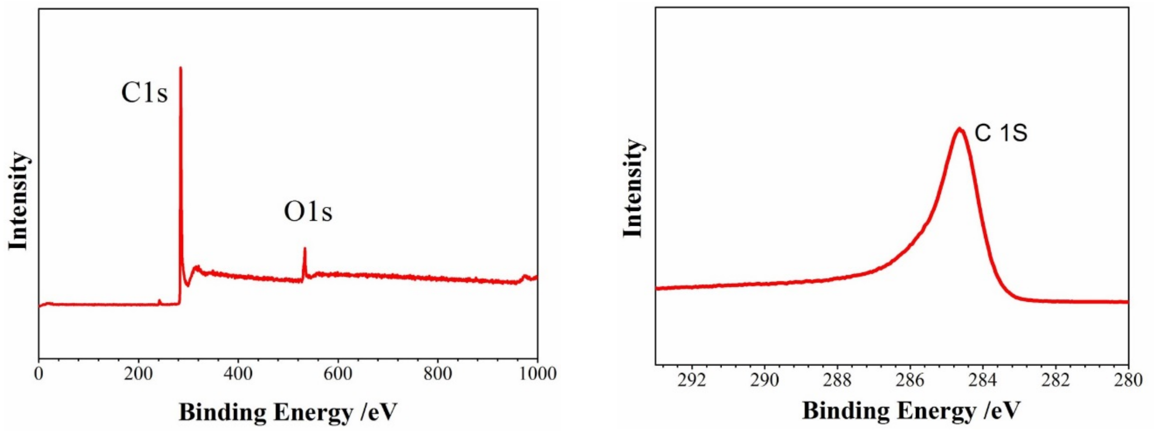

Figure S5. XPS Patterns of Single Layer Graphene. (ACS Material-Graphene Factory)

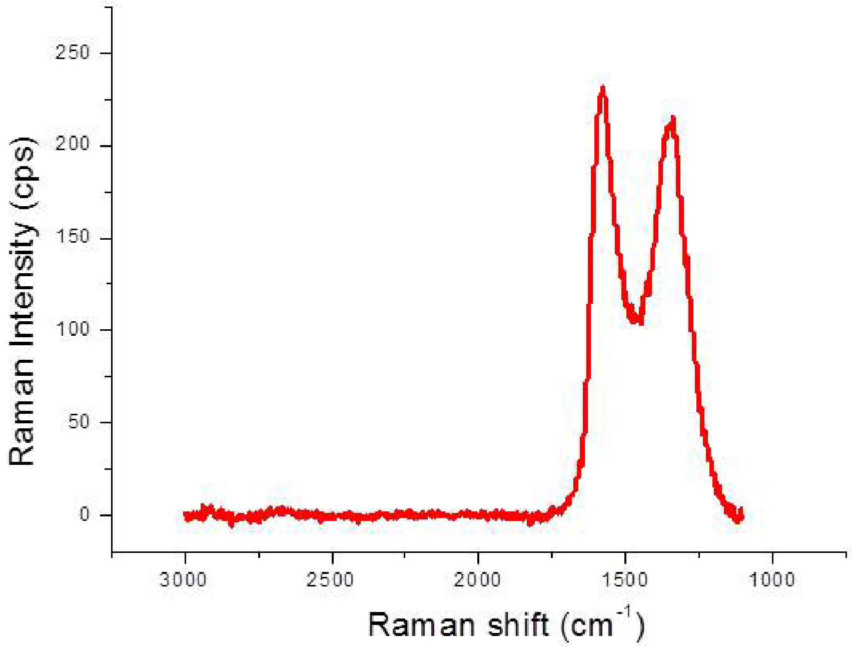

Figure S6. Raman Spectrum of Single Layer Graphene (ACS Material-Graphene Factory)
